# Comparative Effects of Basil Seed Consumption and Endurance Exercise on Irisin, Interleukin 6 (IL-6), Tumor Necrosis Factor-Alpha (TNF-α), and Leptin Levels: A Prospective Observational Study

**DOI:** 10.7759/cureus.75870

**Published:** 2024-12-17

**Authors:** P Adilakshmi, V Suganthi, Suresh Babu Sayana, Senthil Kumar, K. Satyanarayana Rao

**Affiliations:** 1 Department of Research, Vinayaka Mission's Research Foundation (Deemed University), Salem, IND; 2 Department of Physiology, Vinayaka Mission's Kirupananda Variyar Medical College and Hospital, Salem, IND; 3 Department of Pharmacology, Government Medical College and General Hospital, Palwancha, IND; 4 Department of Anatomy, Vinayaka Mission's Kirupananda Variyar Medical College and Hospital, Salem, IND; 5 Departement of General Medicine, Nimra Institute of Medical Sciences, Vijayawada, IND

**Keywords:** basil seeds, endurance exercise, il-6, inflammatory markers, irisin, leptin, metabolic markers, tnf-α

## Abstract

Background and objective

There is growing concern regarding metabolic and inflammatory disorders globally, underscoring the need for effective nonpharmacological interventions. Irisin, interleukin 6 (IL-6), tumor necrosis factor-alpha (TNF-α), and leptin are biomarkers of metabolic and inflammatory health. In this study, we aimed to investigate the comparative effects of basil seed supplementation or endurance exercise on biomarkers of chronic oxidative stress.

Methods

This prospective observational study enrolled 60 participants who were randomly divided into two groups: Group A (basil seeds, n=30) and Group B (endurance exercise, n=30). Participants in Group A consumed 10 grams of soaked basil seeds daily for eight weeks, while those in Group B underwent a supervised endurance exercise regimen (45 minutes/day, five days/week). Biomarkers, including irisin, IL-6, TNF-α, and leptin, were quantified using enzyme-linked immunosorbent assay (ELISA) both at baseline and post-intervention. Within-group and between-group differences were analyzed using paired and independent t-tests, with a significance threshold of p<0.05.

Results

Overall, the baseline characteristics in Group A (age: 35.4 ± 5.0 years; BMI: 24.5 ± 2.3 kg/m²) and B (age: 34.9 ± 4.8 years; BMI: 24.9 ± 2.4 kg/m²) were comparable (p>0.05). Significant changes were observed in both groups post-intervention. Irisin levels increased moderately in group A (47.23 ± 4.8 µg/mL to 50.79 ± 5.13 µg/mL, p<0.01), and IL-6, TNF-α, and leptin decreased (IL-6: 28.2 ± 2.13 pg/mL to 18.1 ± 2.01 pg/mL, p<0.01; TNF-α: 34.5 ± 2.72 pg/mL to 23.6 ± 2.64 pg/mL, p<0.01; and leptin: 8.4 ± 1.96 ng/mL to 3.9 ± 0.83 ng/mL, p<0.01). Group B showed a significant increase in irisin (44.04 ± 3.58 µg/mL to 150.1 ± 9.32 µg/mL, p<0.001) and a non-significant rise in IL-6 (31.65 ± 2.4 pg/mL to 35.64 ± 3.17 pg/mL, p>0.05). TNF-α levels increased in Group B (31.65 ± 3.27 pg/mL vs. 61.66 ± 3.85 pg/mL, p<0.001) and decreased in Group A, while leptin levels slightly decreased in Group B (7.94 ± 1.27 ng/mL vs. 4.19 ± 0.64 ng/mL, p>0.05).

Conclusions

Supplementation with basil seed and exercise training with endurance exercise had distinct effects on biomarkers of metabolism and inflammation. Irisin levels were significantly improved by endurance exercise, while basil seeds were most effective in reducing IL-6, TNF-α, and leptin. The findings suggest that basil seeds and exercise provide synergistic effects against metabolic and inflammatory diseases.

## Introduction

Modern societies are experiencing a steep increase in metabolic and inflammatory disorders such as obesity, diabetes, and cardiovascular diseases. Interestingly, these conditions are strongly linked with chronic low-grade inflammation, dysregulation of metabolic activity, and hormonal abnormalities [1]. Irisin, interleukin-6 (IL-6), tumor necrosis factor-alpha (TNF-α), and leptin are biomarkers that are important in interpreting paths of metabolism and inflammation. In recent years, much attention has been placed on targeting these markers via nonpharmacological interventions such as dietary supplements and physical activity [2].

Ocimum basilicum, commonly known as basil seeds, has great potential as a functional food with several health benefits. Basil seeds provide approximately 480 kcal per 100 g, making them an energy-dense option. They are rich in carbohydrates (42-43 g per 100 g), with a significant portion comprising dietary fiber (37-40 g per 100 g), which contributes to their ability to improve satiety and regulate metabolic health. Also, they are a good source of proteins (14-20 g per 100 g) and fats (10-12 g per 100 g), including 4-6 g of omega-3 fatty acids, which have anti-inflammatory properties. They are also packed with essential minerals such as calcium (300-350 mg), iron (10-12 mg), magnesium (60-70 mg), phosphorus (80-90 mg), and potassium (120-150 mg) per 100 g. Moreover, they provide a modest amount of vitamin C (1-2 mg) and are a rich source of antioxidants, particularly polyphenols, which contribute to their anti-inflammatory and metabolic benefits. This nutritional profile underscores the role of basil seeds as a valuable dietary intervention for promoting metabolic health and reducing inflammation [3]. However, their functions in modulating irisin, IL-6, TNF-α, and leptin have not yet been thoroughly explored. It is known that during physical activity, irisin, a myokine, is released, and it is involved in the promotion of energy expenditure and metabolic regulation [4].

Likewise, leptin, a hormone involved in appetite regulation and energy balance and associated with a variety of metabolic diseases, and other pro-inflammatory cytokines such as IL-6 and TNF-α are essential in the pathophysiology of metabolic diseases [5]. It is well known that endurance exercise, normally used as a strategy to improve metabolic health and decrease systemic inflammation, directly affects these biomarkers in a positive manner [6]. Although evidence exists regarding the benefits of basil seeds and endurance exercise individually [3,6], there is a lack of comparative data evaluating their combined effects on metabolic and inflammatory biomarkers.

This study aims to evaluate the effects of basil seed supplementation and endurance exercise on key biomarkers, including irisin, IL-6, TNF-α, and leptin, which are critical in metabolic regulation and inflammation. By comparing these two interventions, the study seeks to determine their relative efficacy and explore the potential for synergistic benefits in managing metabolic and inflammation-related disorders. We believe our findings could contribute to developing nonpharmacological strategies for addressing chronic conditions associated with metabolic dysfunction and systemic inflammation.

## Materials and methods

Study design and population

A prospective observational study was conducted over eight weeks at the Nimra Institute of Medical Sciences, Vijayawada, Andhra Pradesh, India. All participants provided written informed consent before enrollment.

Baseline assessments

Baseline physical activity levels were assessed using a standardized questionnaire, which also included data on participants' occupations. The occupations reported were categorized based on physical activity intensity levels (e.g., sedentary, moderate, or active), enabling a clearer understanding of the participants' baseline activity patterns. This information was used to ensure that both groups were comparable in terms of physical activity levels and to minimize potential confounding factors related to occupational activity or stress.

Inclusion criteria

The study included 60 healthy adults aged between 18 and 45 years, who were randomly divided into two groups: Group A: basil seeds (n=30); Group B: endurance exercise (n=30). Participants were instructed to maintain their usual dietary habits throughout the study, with no dietary modifications other than the basil seed supplementation provided to Group A. Baseline physical activity levels were assessed and confirmed to be comparable across both groups to ensure uniformity and minimize variability. These considerations were implemented to reduce confounding factors and enhance the reliability of the observed outcomes.

Exclusion criteria

The exclusion criteria for the study were carefully defined to minimize confounding factors related to pro-inflammatory states. Participants with chronic injuries, chronic pain, or any known conditions associated with heightened inflammatory responses, such as autoimmune disorders, were excluded. Additionally, individuals with pre-existing metabolic disorders, chronic inflammatory diseases, or those on anti-inflammatory medications were not included. Although no specific blood tests or radiographic procedures were conducted to screen participants, the exclusion process relied on a detailed medical history and self-reported health status to identify and omit individuals with potential pro-inflammatory conditions. These criteria ensured a more homogeneous study population and enhanced the reliability of the findings.

Group A (basil seeds)

For the entire eight weeks, participants consumed 10 g of dry-weight basil seeds, which were soaked in water for 30 minutes and consumed once daily before breakfast. Participants in the basil seed group continued with their usual daily activities without engaging in any additional exercise-based interventions. This approach ensured that the observed effects in this group could be solely attributed to basil seed supplementation, without the influence of changes in physical activity levels. 

To ensure compliance with the dietary intervention, participants in the basil seed group were provided with pre-measured portions of basil seeds and instructed on their preparation and consumption. Weekly follow-ups were conducted to monitor adherence, and participants were encouraged to report any deviations from the protocol. These measures helped maintain consistency and reliability throughout the study.

Group B (endurance exercise)

Participants in Group B underwent a structured and supervised endurance exercise program, which included moderate activities such as running and cycling. The sessions were conducted for 45 minutes daily, five days a week, over an eight-week period. The exercise regimen was designed to ensure consistency and adherence, with regular monitoring to maintain the intensity and duration of the activities.

Outcome measures

The primary outcomes of the study included the levels of irisin (measured in µg/mL), IL-6 (measured in pg/mL), TNF-α (measured in pg/mL), and leptin (measured in ng/mL). Blood samples were collected in a fasting state in the morning to ensure consistency and minimize variability in biomarker levels due to diurnal fluctuations or recent food intake. Participants were advised to maintain adequate hydration before sample collection but were instructed to avoid consuming any food or beverages other than water for at least eight hours before the procedure. Additionally, all participants were encouraged to follow their usual dietary habits throughout the study period, with no specific modifications apart from the basil seed supplementation in Group A. These measures were implemented to control for potential confounding factors and ensure the reliability of the biomarker analysis. The quantification of these biomarkers was performed using enzyme-linked immunosorbent assays (ELISA), following standard protocols to ensure accuracy and reproducibility [7].

Participant monitoring

Participants were monitored through weekly phone-based check-ins to ensure adherence to the interventions and to address any concerns or adverse events. During these calls, participants provided self-reported updates on compliance, symptoms, and any difficulties related to the interventions.

Data analysis

Data analysis was conducted using SPSS Statistics version 26.0 (IBM Corp., Armonk, NY). The software was employed to perform comprehensive statistical evaluations of the study data. Within-group changes in biomarker levels before and after the interventions were assessed using paired t-tests, which allowed for the identification of significant differences within each group over time. Between-group comparisons were performed using independent t-tests to evaluate differences in outcomes between the basil seed and endurance exercise groups. A p-value <0.05 was considered statistically significant, ensuring robust and reliable conclusions regarding the efficacy of the interventions.

Compliance and monitoring

Weekly self-reports and investigator follow-ups were used to monitor compliance with the interventions among participants. In Group B, the exercise session was supervised to make sure the protocol was adhered to. The adverse events were documented and quickly addressed.

Ethical approval

We obtained relevant institutional ethical committee approval before the study's commencement (approval no: 809/NIMS/Admin/28; dated March 5, 2022). The committee evaluated the study design, protocols, and procedures to confirm that the rights, safety, and well-being of participants were protected. Additionally, the ethics committee played an oversight role throughout the study, monitoring compliance with approved ethical standards to uphold the integrity of the research.

## Results

The study comprised two groups: Group A (basil seeds, n=30) and Group B (endurance exercise, n=30). As detailed in Table 1, baseline characteristics such as age, BMI, weight, height, sex distribution, occupational activity levels, and physical activity levels [measured in metabolic equivalent of task (MET) hours per week] were comparable between the groups, with no statistically significant differences observed (p>0.05). This ensured that the groups were well-matched at the outset, allowing the effects observed during the intervention to be attributed to the respective treatments.

**Table 1:** Baseline characteristics of the study groups The groups were comparable across all parameters, with no statistically significant differences (p>0.05) BMI: body mass index; MET: metabolic equivalent of task; SD: standard deviation

Variable	Group A (basil seeds)	Group B (endurance exercise)	P-value
Sex (male/female), n	15/15	16/14	0.78
Age, years, mean ± SD	35.4 ± 5.0	34.9 ± 4.8	0.84
Weight, kg, mean ± SD	68.2 ± 7.1	69.5 ± 6.8	0.69
Height, cm, mean ± SD	165.4 ± 8.2	166.7 ± 7.9	0.73
BMI (kg/m^2^, mean ± SD	24.5 ± 2.3	24.9 ± 2.4	0.71
Occupation (sedentary/moderate/active), n	10/15/2005	8/12/2010	0.65
Physical activity levels (MET hours/week), mean ± SD	18.5 ± 3.2	19.1 ± 3.5	0.82

The mean age in Group A was 35.4 ± 5.0 years, while it was 34.9 ± 4.8 years in Group B. The mean BMI for Group A was 24.5 ± 2.3 kg/m², and 24.9 ± 2.4 kg/m² for Group B. Group A demonstrated increased irisin levels from a baseline of 47.23 ± 4.8 µg/mL to 50.79 ± 5.13 µg/mL post-intervention. In contrast, Group B demonstrated a significant increase from a baseline value of 44.04 ± 3.58 µg/mL to 150.1 ± 9.32 µg/mL post-intervention (p<0.001, Group B>Group A). Group A showed a significant reduction in IL-6 levels from a baseline level of 28.2 ± 2.13 pg/mL to 18.1 ± 2.01 pg/mL post-intervention. On the other hand, the increase in Group B was not significant: from a baseline value of 31.65 ± 2.4 pg/mL to 35.64 ± 3.17 pg/mL post-intervention. Group A demonstrated a significantly greater reduction in IL-6 levels compared to Group B.

After the intervention, TNF-α levels decreased significantly in Group A: from a baseline value of 34.5 ± 2.72 pg/mL to 23.6 ± 2.64 pg/mL. In contrast, Group B showed a significant increase from a baseline value of 31.65 ± 3.27 pg/mL to 61.66 ± 3.85 pg/mL post-intervention (p<0.001) (Table 2).

**Table 2:** Post-intervention comparisons of metabolic and inflammatory markers between basil seeds and endurance exercise groups IL-6: interleukin-6; TNF-α: tumor necrosis factor-alpha; SD: standard deviation

Parameter	Timeline	Group A (basil seeds), mean ± SD	Group B (endurance exercise), mean ± SD	P-value
Irisin, µg/mL	Baseline	47.23 ± 4.8	44.04 ± 3.58	< 0.001
Irisin, µg/mL	Post-intervention	50.79 ± 5.13	150.1 ± 9.32	< 0.001
IL-6, pg/mL	Baseline	28.2 ± 2.13	31.65 ± 2.4	> 0.05
IL-6, pg/mL	Post-intervention	18.1 ± 2.01	35.64 ± 3.17	< 0.001
TNF-α, pg/mL	Baseline	34.5 ± 2.72	31.65 ± 3.27	< 0.001
TNF-α, pg/mL	Post-intervention	23.6 ± 2.64	61.66 ± 3.85	< 0.001
Leptin, ng/mL	Baseline	8.4 ± 1.96	7.94 ± 1.27	> 0.05
Leptin, ng/mL	Post-intervention	3.9 ± 0.83	4.19 ± 0.64	< 0.001

Group A (basil seeds) demonstrated a significant reduction in leptin levels, decreasing from a baseline value of 8.4 ± 1.96 ng/mL to a post-intervention value of 3.9 ± 0.83 ng/mL, highlighting the impact of basil seed supplementation on reducing this biomarker. In contrast, Group B (endurance exercise) exhibited only a modest decrease in leptin levels from 7.94 ± 1.27 ng/mL at baseline to 4.19 ± 0.64 ng/mL post-intervention; this change was not statistically significant (p>0.05). These findings suggest that basil seeds may have been more effective in modulating leptin levels compared to endurance exercise over the study period.

Adverse events and patient experiences were closely monitored throughout the study to ensure participant safety. In the basil seed group, participants were asked to report any discomfort, such as nausea, bloating, or swallowing difficulties, as 10 grams of basil seeds, after swelling, could potentially cause such issues. A few participants reported mild bloating during the first week, but this resolved without requiring any intervention, and no serious adverse events were noted. In the endurance exercise group, participants were monitored for joint or muscle injuries throughout the intervention. During the second phlebotomy, participants were screened for any signs of inflammation, discomfort, or injuries. None of the participants reported injuries or symptoms suggesting elevated pro-inflammatory cytokines due to physical activity. These findings indicate that both interventions were well-tolerated by the participants.

## Discussion

In this study, the metabolic and inflammatory biomarkers irisin, IL-6, TNF-α, and leptin were analyzed to evaluate the effects of basil seed supplementation and endurance exercise. Both interventions significantly influenced these biomarkers, with basil seeds causing pronounced reductions in inflammatory markers and endurance exercise markedly increasing irisin levels [8,9]. Endurance exercise promotes the release of irisin, a myokine that enhances energy expenditure, improves metabolic regulation through mitochondrial function, and induces the browning of white adipose tissue. Exercise-induced muscle contractions also trigger a transient rise in pro-inflammatory cytokines like IL-6 and TNF-α, reflecting an acute inflammatory response [9,10]. However, regular physical activity leads to long-term adaptations that reduce systemic inflammation by activating anti-inflammatory pathways and improving cytokine sensitivity. This dual role of exercise highlights its therapeutic potential for managing metabolic and inflammatory disorders.

Irisin levels showed a significant increase following endurance exercise, while the increase observed with basil seed treatment was modest in comparison. This finding therefore corroborates the role of physical activity in increasing irisin, a myokine with a clear, important role in the energetics and metabolism control [10,11]. However, the antioxidant properties of basil seeds may indirectly influence muscle function and metabolism, as reflected in the modest increase in irisin in the basil seed group [12]. These results indicate that endurance exercise is the more effective intervention for increasing irisin levels and consequently enhancing metabolic health [13].

Routine blood tests, including complete blood count (CBC), erythrocyte sedimentation rate (ESR), and C-reactive protein (CRP), were performed during the initial screening and post-intervention assessments to monitor inflammatory and acute reactive states. Although these tests were not part of the primary outcomes, they provided valuable information on the overall hematological and inflammatory profiles of the participants. The data from these tests showed no significant abnormalities or unexpected changes across the groups, further supporting the safety of the interventions. 

In Group A (basil seeds), both IL-6 and TNF-α levels significantly decreased, indicating a strong anti-inflammatory effect. Conversely, Group B (endurance exercise) showed an unexpected increase in IL-6 levels and a significant rise in TNF-α levels, likely reflecting an acute inflammatory response induced by the exercise intervention. This contrast in outcomes suggests that basil seeds have a strong anti-inflammatory power because of their polyphenol and antioxidant content, and endurance exercise may temporarily elevate inflammatory markers due to acute systemic inflammation induced by intense physical activity [14,15].

Leptin levels significantly decreased in the basil seed group, demonstrating a notable effect of the intervention; however, the decrease observed in the exercise group was not statistically significant, suggesting a less pronounced impact. The high dietary fiber of basil seeds might modulate leptin levels by satiating and improving metabolic profiles [16]. Interestingly, the minimal reduction of leptin in the exercise group indicates that endurance exercise mainly impacts leptin sensitivity rather than decreasing leptin levels by itself [17]. 

These findings indicate that the consumption of basil seeds could serve as a dietary intervention for lowering inflammatory biomarkers and hold more potential for increasing irisin levels when compared to endurance exercise. Basil seeds constitute a convenient and available source of treatment for systemic inflammation; exercise, on the other hand, has wider metabolic actions. Combining the approaches may have complementary benefits: they may help regulate metabolism, and they may attenuate inflammation.

Limitations

The observational design did not allow us to draw causal inferences about how interventions cause outcomes. Findings cannot be generalized with their general population because of the small sample size. The study duration of eight weeks may be insufficient to fully capture the long-term effects of basil seeds or endurance exercise on metabolic and inflammatory biomarkers. Additionally, the unexpected increase in IL-6 and TNF-α in the exercise group likely reflects acute rather than chronic changes, warranting further investigation. Although stress levels were not directly measured, their potential influence on serum biomarkers is acknowledged as a limitation of the study. Adherence to basil seed consumption was self-reported, which could introduce bias. Future randomized controlled trials with larger cohorts and extended durations are needed to validate these findings.

## Conclusions

This study uniquely evaluated the effects of basil seed supplementation and endurance exercise on metabolic and inflammatory biomarkers. Basil seed supplementation demonstrated significant anti-inflammatory effects, reducing levels of key inflammatory markers such as IL-6, TNF-α, and leptin. Endurance exercise, on the other hand, markedly increased circulating irisin levels, highlighting its role in enhancing metabolic regulation. However, the exercise intervention was also associated with an acute rise in inflammatory markers, likely reflecting a transient response to physical activity. These findings suggest that basil seeds could serve as a potent dietary intervention for reducing systemic inflammation, while endurance exercise may optimize metabolic health. Combining these interventions may provide complementary benefits, offering a holistic approach to managing metabolic and inflammatory disorders.
